# Diagnostic Accuracy of McMurray’s Test for Meniscal Injury of the Knee Joint, Taking Arthroscopy as the Gold Standard

**DOI:** 10.7759/cureus.83970

**Published:** 2025-05-12

**Authors:** Luqman Khan, Qazi Shahrukh, Arsalan Shah Roghani, Salma Ghaffar, Rao E Hassan, Syed Suleman Shah, Haris Khan, Ismail S Tarangi, Noormal, Aftab Ali

**Affiliations:** 1 Orthopedics and Trauma, Khyber Teaching Hospital Medical Teaching Institution (MTI), Peshawar, PAK; 2 Surgery, Poonch Medical College, Rawalakot, PAK; 3 General Surgery, District Headquaters Hospital, Pishin, PAK; 4 Surgery, Ayub Teaching Hospital Medical Teaching Institution (MTI), Abbottabad, PAK; 5 Surgery, Khyber Medical College, Peshawar, PAK

**Keywords:** arthroscopy, diagnostic arthroscopy, diagnostic efficacy, magnetic resonance imaging, mcmurray’s test, meniscal injury, meniscal tear, the knee joint

## Abstract

Introduction

Meniscal tears are a common cause of knee pain and disability. While McMurray’s test is widely used for clinical diagnosis, its accuracy varies across studies. This research aimed to assess the diagnostic accuracy of McMurray’s test for detecting meniscal tears, using arthroscopy as the gold standard.

Materials and Methods

This prospective observational study included 152 patients aged 20-40 years. McMurray’s test was performed on all patients by an experienced orthopedic surgeon. Arthroscopy was later conducted for confirmation. Sensitivity, specificity, positive predictive value, negative predictive value, diagnostic accuracy, and receiver-operating characteristic (ROC) area under the curve (AUC) were calculated for both menisci separately.

Results

Out of 152 patients, McMurray’s test was positive in 57.9%, and arthroscopy confirmed meniscal tears in 61.2% of cases. The test showed an accuracy of 61.2% and 91.5% for diagnosing medial and lateral meniscus tears, respectively. ROC analysis for the medial and lateral meniscus demonstrated an AUC of 61.2% and 75.9%, respectively.

Conclusion

McMurray’s test demonstrated modest diagnostic accuracy in detecting meniscal injuries. While useful as an initial clinical tool, it should be interpreted with caution, and further clinical tests and imaging may be warranted for definitive diagnosis.

## Introduction

Meniscal tears are among the most common knee injuries. McMurray’s test and joint line tenderness remain widely used diagnostic tools, yet their reported accuracy varies considerably across studies [1]. This inconsistency can complicate clinical decision-making, especially when evaluating the necessity of additional imaging before proceeding to arthroscopy, which remains the diagnostic gold standard.

The clinical diagnosis of meniscal tears largely depends on the examiner’s expertise. Pain is usually localized to the medial or lateral joint line, with traumatic injuries often occurring during weight-bearing knee flexion combined with rotational stress. An audible “pop” may or may not be present. Symptoms typically worsen with flexion or loading, and patients frequently report mechanical symptoms such as locking or a distinct “clunk” during movement [2]. Importantly, in patients with moderate-to-severe osteoarthritis, arthroscopy has not demonstrated significant benefit over-optimized non-operative therapy [3].

Palmanovich et al. prospectively compared preoperative clinical diagnoses of meniscal and ACL injuries to arthroscopic findings in 753 patients [4]. Arthroscopy confirmed 65% of medial meniscal tears and 54% of lateral tears, prompting the authors to advocate for supplemental imaging when isolated meniscal injuries are suspected [4]. Antinolfi et al. prospectively compared clinical examination, MRI, and arthroscopy in 80 patients with suspected medial meniscal tears. Clinical assessment demonstrated higher diagnostic performance than MRI, with greater sensitivity (91% vs. 85%), specificity (87% vs. 75%), accuracy (90% vs. 82%), positive predictive value (PPV) (94% vs. 88%), and negative predictive value (NPV) (81% vs. 71%). Based on these findings, the authors recommended the use of MRI only as an additional diagnostic tool after clinical examination [5]. Nevertheless, MRI remains the most accurate non-invasive modality for evaluating meniscal pathology, although arthroscopy continues to serve as the gold standard [6].

Hing et al. reported a wide range of sensitivity for the medial (16-88%) and lateral (25-79%) meniscus for McMurray’s test and its modified variants [7]. Ercin et al. prospectively compared clinical examination and MRI with arthroscopic findings in diagnosing medial meniscal tears [8]. Clinical evaluation by experienced surgeons showed higher specificity (90% vs. 60%), PPV (95% vs. 83%), NPV (90% vs. 86%), and overall accuracy (93% vs. 83%) compared to MRI. The authors concluded that when multiple meniscal tests, including McMurray’s, are performed by experienced clinicians, diagnostic accuracy is high enough to reduce reliance on MRI [8]. Gupta et al. evaluated McMurray’s test for detecting medial meniscal tears, reporting a sensitivity of 50%, specificity of 79%, PPV (68%), NPV (67.5%), and overall diagnostic accuracy of 67.74% [1].

There is a lack of regional data on the diagnostic accuracy of McMurray's test for meniscal injuries of the knee joint, and very few studies have been conducted on diagnostic accuracy in our setting. This study aims to evaluate the effectiveness of the McMurray's test in diagnosing medial meniscal tears, using arthroscopic examination as the definitive reference standard, and contribute valuable insights for orthopedic surgeons in the region, guiding them in forming differential diagnoses when evaluating patients presenting with knee symptoms.

## Materials and methods

This prospective observational study was conducted in the Department of Orthopedics at Khyber Teaching Hospital, Peshawar, from October 2022 to April 2024. A total of 152 patients were included. The sample size was calculated using the WHO sample size calculator based on a meniscal injury prevalence of 68.7% [8], a sensitivity of 54% [1], a specificity of 79% [1], a 95% confidence interval, and a 6% margin of error.

Non-probability consecutive sampling was employed. Patients aged 20 to 40 years presenting with knee pain lasting more than three weeks, giving way, a visual analog scale pain score greater than 5, or clinical swelling in the affected knee for over three months compared to the contralateral side were included. Both male and female patients were eligible. Only patients classified as Grade I or Grade II under the American Society of Anesthesiology physical status classification were enrolled. Exclusion criteria included systemic diseases associated with knee pain, open knee fractures, Kellgren-Lawrence grade III and IV osteoarthritis, rheumatoid arthritis, multi-ligament injury cases, previous history of knee surgery, and a body mass index (BMI) greater than 30.

Following ethical approval, patients were recruited from the outpatient department. Ethical approval was provided by the Khyber Medical College Institutional Research and Ethical Review Board (IREB) (Approval No. 716/ARC/KMC). Written informed consent was obtained prior to enrollment. A detailed clinical history was recorded, and a comprehensive physical examination of the knee, including special joint stability tests, was conducted by an experienced orthopedic surgeon to make a differential diagnosis and exclude non-meniscal injuries. McMurray’s test was performed on all patients. The McMurray’s test was considered positive if the patient experienced pain and/or an audible or palpable click, clunk, or snap during the maneuver. Subsequently, an arthroscopic examination was scheduled and performed by a specialist orthopedic surgeon trained in sports-related surgeries and arthroscopy. The arthroscopy findings were used as the gold-standard test to diagnose meniscal injuries [9].

Demographic and clinical variables, including age, gender, height, weight, BMI, and duration of symptoms, were documented. The outcomes of McMurray’s test and arthroscopic findings were recorded for each patient. Means and standard deviations were calculated for continuous variables, while frequencies and percentages were used for categorical data. Diagnostic accuracy, sensitivity, specificity, PPV, and NPV were calculated using a 2x2 contingency table. Statistical analysis of sensitivity and specificity was performed using the McNemar test for correlated proportions. All tests were two-sided, evaluating the hypothesis that the diagnostic performance of McMurray’s test differed from that of arthroscopic examination. A p-value of less than 0.05 was considered statistically significant. Stratification was performed based on age, gender, injury type, residence, socioeconomic status, symptom duration, and BMI to control for effect modifiers. Post-stratification analysis was conducted to assess diagnostic accuracy, sensitivity, specificity, PPV, and NPV across subgroups. Ninety-five percent likelihood confidence intervals (CI) were analyzed for these metrics. To evaluate the diagnostic accuracy of McMurray’s test, receiver-operating characteristic (ROC) curves were plotted, comparing sensitivity and specificity based on the arthroscopic findings of medial or lateral meniscal injuries.

## Results

**Figure 1:**
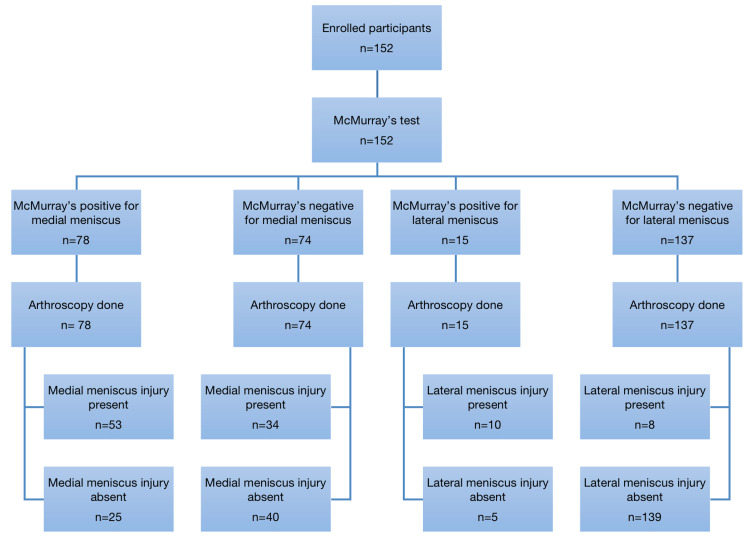
Flow diagram of the study. "n" represents the number of participants.

A total of 152 patients were enrolled in the study. The baseline characteristics of the participants are given in Table 1, while Figure 1 shows the flow of patients through the study.

**Table 1:** Baseline characteristics of the study population. N: number of cases; SD: standard deviation; kgs: kilograms; BMI: body mass index; RTA: road traffic accidents. The data has been represented as N (%), mean±SD.

Characteristics		N (%), mean±SD
Enrolled cases		152 (100%)
Gender	Male	103 (67.8%)
	Female	49 (32.3%)
Age (years)		29 ± 5.72
Weight (kgs)		72 ± 9.91
Height (meters)		1.7 ± 0.07
BMI (kg/m²)		26.4 ± 3.69
Duration of symptoms (months)		10 ± 4.58
Age groups (years)	20–25	46 (30.3%)
	26-30	44 (28.9)
	31-35	36 (23.7%)
	36-40	26 (17.1%)
Mechanism of injury	RTA	85 (55.9%)
	Fall from height	43 (28.3%)
	Sports-related	25 (16.5%)
Residential status	Rural	87 (57.3)
	Urban	65 (42.7%)
Socioeconomic status	Lower-income group	47 (30.9%)
	Middle-income group	51 (33.6%)
	Higher-income group	54 (35.5%)

Meniscal tears identified on McMurray’s test and confirmed on arthroscopy were stratified according to age, gender, mechanism of injury, residence, socioeconomic status, BMI, and duration of symptoms (see Appendices). On clinical assessment using McMurray’s test, 57.9% (n=88) were positive for meniscal tears overall, while 42.1% (n=64) tested negative. The arthroscopic evaluation showed that 61.2% (n=93) of patients had meniscal tears, whereas 38.8% (n=59) did not. The medial meniscus was injured in 51.3% (n=78) and 57.2% (n=87) on McMurray’s test and arthroscopy, respectively, while the lateral meniscus was injured in 9.9% (n=15) and 11.8% (n=18) on McMurray’s test and arthroscopy, respectively.

Based on the 2×2 contingency table, the diagnostic accuracy of McMurray’s test was calculated using arthroscopy as the gold standard. The sensitivity, specificity, PPV, NPV, and diagnostic accuracy of McMurray’s test for both lateral and medial menisci are shown (Tables 2, 3).

**Table 2:** A 2×2 table to find diagnostic accuracy of McMurray's test. χ²: chi-square test statistics for McNemar test for correlated proportions; p<0.05 considered significant.

	Meniscal injury (tear) on arthroscopy			χ²	P-value
Medial meniscal injury (tear) on McMurray's test		+	-	1.37	0.24
	+	53 (34.9%)	25 (16.5%)		
	-	34 (22.4%)	40 (26.3%)		
Lateral meniscal injury (tear) on McMurray's test	+	10 (6.6%)	5 (3.3%)	0.69	0.40
	-	8 (5.3%)	129 (84.9%)		

**Table 3:** Diagnostic test accuracy metrics for McMurray's test. 95% CI: 95% confidence interval lower and upper limit; χ²: wald chi-square test statistics for 95% CI; PPV: positive predictive value; NPV: negative predictive value; p<0.05 considered significant.

McMurray's test	Medial meniscus injury (95% CI)	χ² (p-value)	Lateral meniscus injury (95% CI)	χ² (p-value)
Sensitivity	60.9% (50.5%, 70.7%)	135.6 (<0.0001)	55.6% (33%, 76.6%)	22.5 (<0.0001)
Specificity	61.5% (49.4%, 72.8%)	104 (<0.0001)	96.3% (92.2%, 98.6%)	3457.2 (<0.0001)
PPV	67.9% (57.1%, 77.6%)	165.3 (<0.0001)	66.7% (41.5%, 86.5%)	30 (<0.0001)
NPV	54.1% (42.7%, 65.1%)	87.1 (<0.0001)	94.2% (89.4%, 86.3%)	2209.1 (<0.0001)
Diagnostic accuracy	61.2%		91.5%	

**Figure 2:**
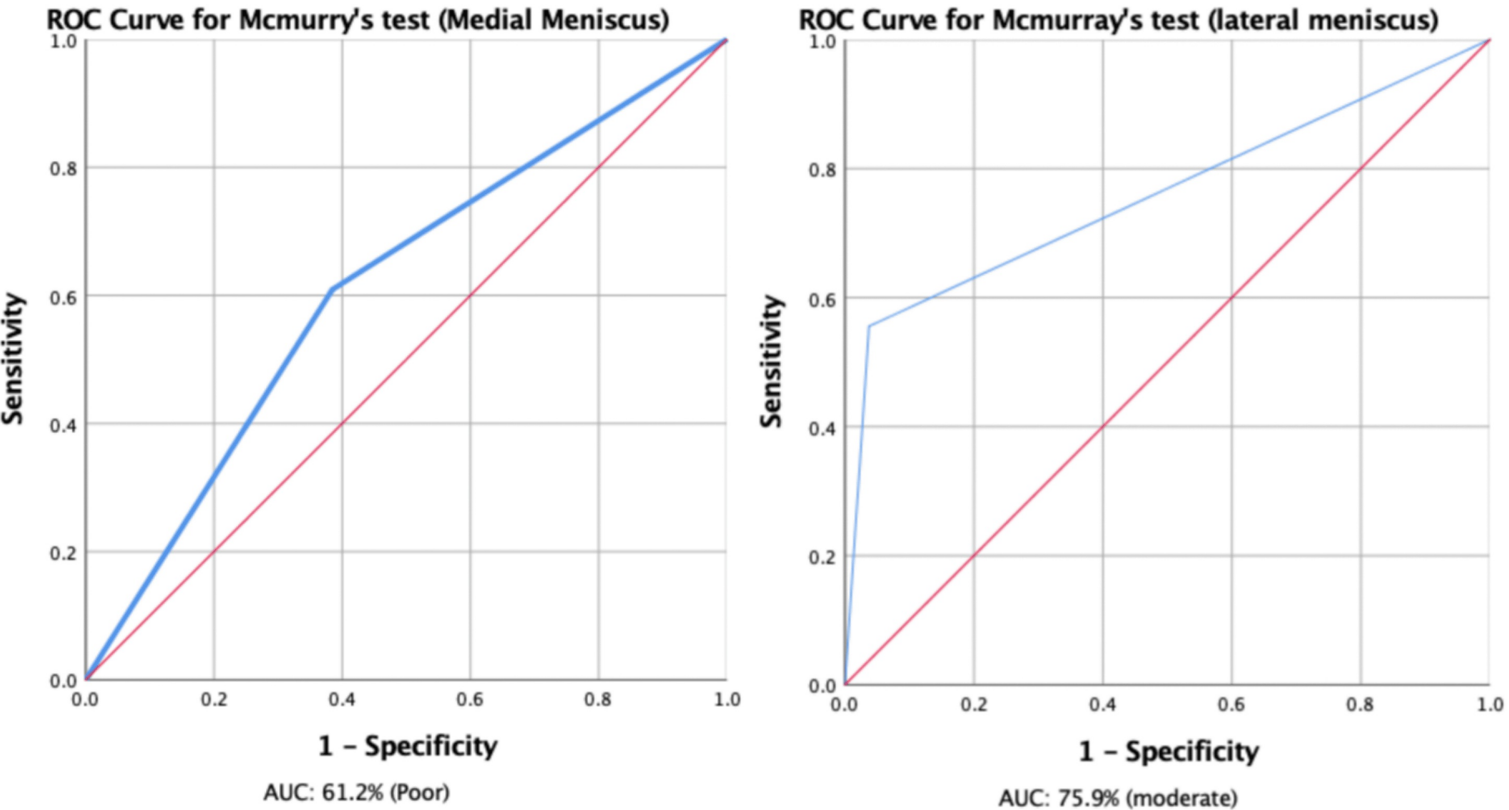
ROC curve of Mcmurray's test for medial and lateral meniscus. ROC: receiver operating characteristic; AUC: area under the curve.

The ROC curve demonstrated an area under the curve of 61.2% (52.2%, 70.3%) and 75.9% (61.3%, 90.5%) for the medial and lateral meniscus, respectively (Figure 2).

Post-stratification analysis for sub-categories revealed that the age group 31-35 years had the highest sensitivity (82.6%), specificity (76.9%), NPV (71.4%), and diagnostic accuracy (80.5%), while sports-related injuries had the highest PPV (86.7%) (Table 4).

**Table 4:** Post-stratification diagnostic test accuracy metrics for McMurray's test for age group 31-35 years and sports related injuries. 95% CI: 95% confidence interval lower and upper limit; χ²: wald chi-square test statistics for 95% CI; PPV: positive predictive value; NPV: negative predictive value; p<0.05 considered significant.

	McMurray's test	Meniscus injury ( 95% CI)		χ² (p-value)
Sports-related injuries	Sensitivity	76.5% (53.5%, 92%)		55.2 (<0.01)
	Specificity	75% (40.9%, 69.3%)		24 (<0.01)
	PPV	86.7% (64.2%, 97.7%)		97.5 (<0.01)
	NPV	60% (30%, 85.4%)		15 (<0.01)
	Diagnostic accuracy	76%		
Age group 31-35 years	Sensitivity	82.6% (64.1%, 94.2%)	109.3 (<0.01)	
	Specificity	76.9% (50.5%, 93.7%)	43.3(<0.01)	
	PPV	86.4% (68.3%, 96.4%)	139.3 (<0.01)	
	NPV	71.4 % (45.5%, 90.1%)	35 (<0.01)	
	Diagnostic accuracy	80.5%		

## Discussion

Meniscal tears typically result from acute trauma or degenerative changes within the meniscal tissue. Patients usually complain about a sudden onset of sharp pain after a twisting injury, mostly with the knee flexed and the foot planted [2,3,5]. Although the pain may be relieved initially, localized discomfort in the affected compartment often remains. Recurrent joint effusions are observed frequently, and patients also report locking symptoms. On physical examination, joint-line tenderness is consistently there, along with a palpable click or snap. Range of motion may be limited due to mechanical obstruction caused by a displaced meniscal fragment [5-7].

Clinicians use various provocative tests to assess meniscal pathology by reproducing the patient’s symptoms. These include palpation-based assessments such as the Bragard, McMurray, Thessaly, and Steinmann II tests, and rotation-based maneuvers like the Apley and Böhler tests. McMurray's test remains one of the most widely used clinical tools. Medial meniscus evaluation involves passive movement of the knee from flexion to extension while applying external tibial rotation. In contrast, lateral meniscal assessment follows the same arc of motion with internal tibial rotation [8,10].

Reported diagnostic accuracy rates range from 0% to 95% for clinical meniscal tests, which are typically performed in non-weight-bearing positions [5,6,10,11]. However, meniscal symptoms commonly present while the patient is weight-bearing. So these tests do not reproduce the mechanisms that produce symptoms in torn menisci [10,11]. It is quite a challenge to diagnose meniscal pathology through these tests. Evidence-based guidelines published in 2003 stated joint-line tenderness as the only reliable clinical test of meniscal injury [12]. While McMurray's test and joint-line tenderness are commonly used, their diagnostic accuracy varies significantly across studies [3-5,10]. This inconsistency complicates decisions regarding the need for advanced imaging, such as MRI, before proceeding with diagnostic arthroscopy, which is both the gold standard and the most frequently performed knee surgery [13,14].

McMurray’s test demonstrates considerable variability in diagnostic accuracy, primarily due to differences in study populations and variations in test methodology [1,15,16]. Recent studies suggest that modifications to the original technique enhance its diagnostic validity [11,15-17]. In this study, McMurray’s test exhibited an accuracy of 61.2%, with sensitivity at 60.9%, specificity at 61.5%, PPV at 67.9%, and NPV at 54% for medial meniscus tear, while those for lateral meniscus were 91.5%, 55.6%, 96.3%, 66.7%, and 94.2%, respectively. Similar to the presented study, Akseki et al. also reported that lateral meniscal tears were diagnosed more accurately than medial meniscal tears, with an overall accuracy of 82% and 66%, respectively [11], while Karachalios et al. found a much higher accuracy (78%) and specificity (94%) for medial meniscal tears, though sensitivity was lower at 48%, while accuracy and specificity for lateral meniscal tears were 84% and 86%, respectively [10]. Similarly, Sae-Jung et al. reported a sensitivity of 70% and specificity of 60.7% for medial meniscal tears, and those of the lateral meniscus were 68.2% and 47.8%, respectively [18].

Discrepancies among studies may result from methodological variations, such as differing definitions of a positive test, which may be based on pain alone or the presence of a medial thud. As Gupta et al. emphasized, the reliability of a diagnostic test depends on its performance, interpretation, and the criteria used to define a positive result [1]. These criteria-including pain, clicks, thuds, or clunks-have varied significantly across studies, contributing to inconsistencies in reported outcomes [7,19]. The studies that include both pain and a click as a positive test [11,18], like the presented study, have higher diagnostic values as compared to studies that only use one or the other of these signs as criteria [19].

This study has several limitations that may impact the generalizability. First, the use of non-probability consecutive sampling could introduce selection bias. Secondly, the study's single-center design may limit the applicability of the results. Furthermore, the lack of blinding during McMurray’s test and arthroscopy may give rise to observer bias.

In summary, this study shows that McMurray’s test lacks high sensitivity for detecting both medial and lateral meniscal injuries, often failing to identify many positive cases. However, the test demonstrates high specificity, NPV, and accuracy for lateral meniscal injuries, making it a reliable tool for those who test negative.

## Conclusions

In conclusion, McMurray’s test demonstrates only a moderate diagnostic utility for medial meniscal tears. However, it shows strong diagnostic accuracy for lateral meniscal tears, despite its low sensitivity. These findings highlight the importance of using other clinical tests and additional imaging tools, such as MRI, collectively to improve diagnostic accuracy. While these results clear some clinical understanding, future studies with larger and more diverse cohorts and multimodal diagnostic approaches are needed to validate these observations.

## Appendices

**Table 5:** Stratification of research categories for McMurray's test and arthroscopy. Data represented as "n"; BMI: body mass index; RTA: road traffic accidents.

Category	Sub-category (n)	McMurray’s test (n)		Arthroscopy (n)	
		Positive (n=88)	Negative (n=64)	Positive (n=93)	Negative (n=59)
Age groups (years)	20-25 (46)	26	20	27	19
	26-30 (44)	25	19	26	18
	31-35 (36)	22	14	23	13
	36-40 (26)	15	11	17	09
Gender	Male (103)	61	42	63	40
	Female (49)	27	22	30	19
Nature of injuries	RTA (85)	48	26	51	33
	Falls related injuries (43)	25	18	25	18
	Sports-related injuries (25)	15	10	17	08
Residential Status	Rural (87)	50	37	53	34
	Urban (65)	38	27	40	25
Socioeconomic status	Poor (47)	28	19	28	19
	Middle class (51)	30	21	33	18
	Rich (54)	30	24	32	22
BMI (kg/m^2^)	< 18.5 (23)	13	10	15	8
	18.5-24.9 (58)	35	23	36	22
	25-29-9 (71)	40	31	42	29
Duration of symptoms (months)	< 6 (19)	10	9	12	7
	6-12 (101)	61	40	63	38
	>12 (32)	17	15	18	14
